# Diaqua­bis­[8-ethyl-5-oxo-2-(piperazin-4-ium-1-yl)-5,8-dihydro­pyrido[2,3-*d*]pyrimidine-6-carboxyl­ato]copper(II) bis[4-(4-carboxyphenoxy)benzoate]

**DOI:** 10.1107/S1600536811006672

**Published:** 2011-02-26

**Authors:** Dian-Zhen Sun, Guang-Ju Zhang, Hai-Yan Chen, Jiang-Hong He, Shi-Wei Yan

**Affiliations:** a College of Chemistry and Chemical Engineering, Southwest University, Chongqing 400715, People’s Republic of China

## Abstract

In the title compound, [Cu(C_14_H_17_N_5_O_3_)_2_(H_2_O)_2_](C_14_H_9_O_5_)_2_, the Cu^2+^ atom, located on an inversion centre, exhibits a distorted octa­hedral geometry, coordinated by four O atoms from two pipemidic acid ligands in equatorial positions and two water mol­ecules in axial positions. The pipemidic acid ligand acts a bidentate ligand and the single deprotonated 4,4′-oxydibenzoic acid acts as an anion. Classical N—H⋯O and O—H⋯O hydrogen bonds are present in the crystal structure.

## Related literature

For general background to the use of quinolones in the treatment of infections, see: Mizuki *et al.* (1996 ▶).
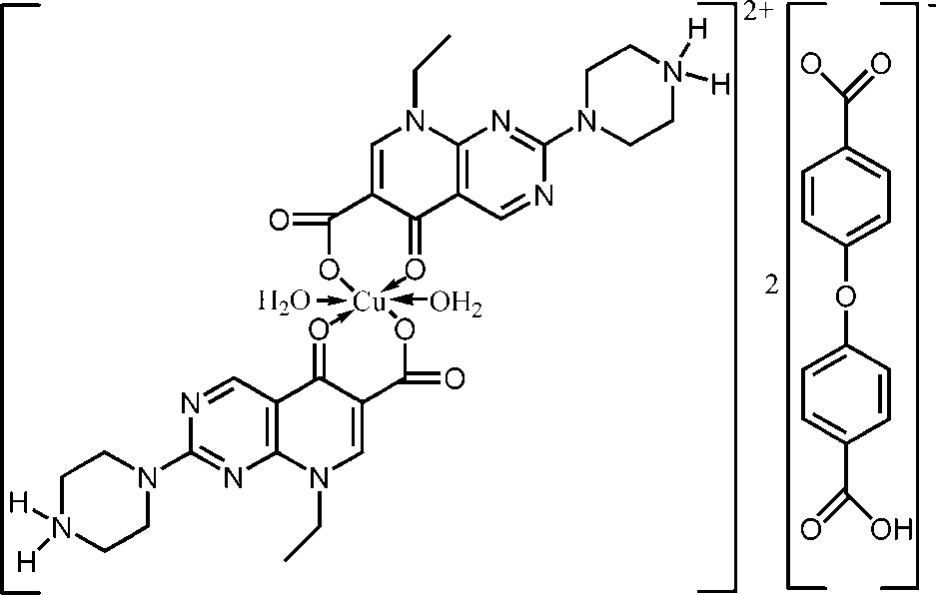


## Experimental

### 

#### Crystal data

[Cu(C_14_H_17_N_5_O_3_)_2_(H_2_O)_2_](C_14_H_9_O_5_)_2_
*M* *_r_* = 1220.66
Triclinic, 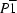

*a* = 8.611 (8) Å
*b* = 12.555 (12) Å
*c* = 13.436 (12) Å
α = 76.222 (10)°
β = 73.299 (10)°
γ = 81.015 (10)°
*V* = 1345 (2) Å^3^
*Z* = 1
Mo *K*α radiation
μ = 0.49 mm^−1^
*T* = 295 K
0.47 × 0.41 × 0.33 mm

#### Data collection

Bruker SMART CCD diffractometer
Absorption correction: multi-scan (*SADABS*; Sheldrick, 1996 ▶) *T*_min_ = 0.801, *T*_max_ = 0.854
11977 measured reflections
5790 independent reflections
4801 reflections with *I* > 2σ(*I*)
*R*_int_ = 0.086

#### Refinement

*R*[*F* ^2^ > 2σ(*F* ^2^)] = 0.049
*wR*(*F* ^2^) = 0.140
*S* = 1.04
5790 reflections
397 parameters
3 restraints
H atoms treated by a mixture of independent and constrained refinement
Δρ_max_ = 0.56 e Å^−3^
Δρ_min_ = −1.01 e Å^−3^

### 

Data collection: *SMART* (Bruker, 2001 ▶); cell refinement: *SAINT* (Bruker, 2001 ▶); data reduction: *SAINT*; program(s) used to solve structure: *SHELXS97* (Sheldrick, 2008 ▶); program(s) used to refine structure: *SHELXL97* (Sheldrick, 2008 ▶); molecular graphics: *SHELXTL* (Sheldrick, 2008 ▶); software used to prepare material for publication: *SHELXL97*.

## Supplementary Material

Crystal structure: contains datablocks I, global. DOI: 10.1107/S1600536811006672/rk2260sup1.cif

Structure factors: contains datablocks I. DOI: 10.1107/S1600536811006672/rk2260Isup2.hkl

Additional supplementary materials:  crystallographic information; 3D view; checkCIF report

## Figures and Tables

**Table 1:** Hydrogen-bond geometry (Å, °)

*D*—H⋯*A*	*D*—H	H⋯*A*	*D*⋯*A*	*D*—H⋯*A*
O*W*1—H*W*1*A*⋯O4^i^	0.85 (1)	2.08 (1)	2.921 (3)	169 (2)
O*W*1—H*W*1*B*⋯O7^ii^	0.85 (1)	2.08 (3)	2.892 (3)	161 (2)
O6—H6*A*⋯O5^iii^	0.81 (3)	1.81 (3)	2.582 (3)	160 (3)
N1—H1*A*⋯O1^ii^	0.90	1.91	2.783 (3)	162
N1—H1*B*⋯O4^iv^	0.90	1.73	2.604 (3)	164

Symmetry codes: (i) 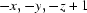; (ii) 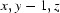; (iii) 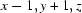; (iv) 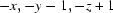.

**Fig. 1.**
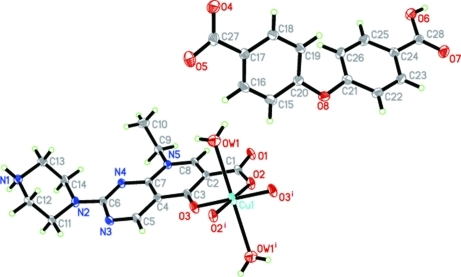
Asymmetric unit of title compound with the atom numbering scheme. Displacement ellipsoids are drawn at 50% probability level. H atoms are presented as a small spheres of arbitrary radius. Symmetry code: (i) -x, -y, -z.

## supplementary crystallographic information

### Comment

Pipemidic acid (H*ppa*, C_14_H_17_N_5_O_3_, 8-ethyl-5,8-dihydro-5- oxo-2-(1-piperazinyl)-pyrido(2,3-*d*)-pyrimidine-6-carboxylic acid) is member of a class of quinolones used to treat infections (Mizuki *et al.*, 1996). H_2_*oba* (4,4'-oxybisbenzoic acid) acts a anion in this complex. The metal complexes of the H*ppa*  and H_2_*oba* have not been reported till; the title copper(II) complex, I, is presented here (Fig. 1).

The Cu atom exhibits an approximate square environment with atoms O2, O3, O2^i^, O3^i^ (see Fig. 1 for symmetry code) of two H*ppa* ligands (two O, O-bidentate). The Cu—O bond distances arising from the two carbonyl oxygen atoms O3 are longer (1.9605 (19)Å) than those arising from the carboxylate oxygen atoms O2 (1.932 (2)Å). The bond angles O2—Cu1—O3^i^ and O2—Cu1—O3 open up slightly from 88.51 (9)° to 91.49 (9)°. The Cu^II^ atom at each short edge of the rectangle are bridged by an water molecule, which also interacts weakly (Cu1···OW1 = 2.642 (10)Å) with the central metal, resulting in a distortion octahedral geometry. Classical N—H···O and O—H···O hydrogen bonds are present in the crystal structure (Table 1).

### Experimental

A mixture of CuI (0.095 g, 0.5 mmol), H*ppa* (0.089 g, 0.25 mmol), H_2_*oba* (0.0645 g, 0.25 mmol) and water (8 ml) was stirred for 30 min in air. The mixture was then transferred to a 18 ml teflon-lined hydrothermal bomb. The bomb was kept at 393 K for 120 h under autogenous pressure. Upon cooling, blue block of I were obtained from the reaction mixture.

### Refinement

The H atoms bonded to C atoms were positioned geometrically and refined using a riding model approximation [aromatic C—H = 0.93Å, aliphatic C—H = 0.96Å–0.97Å], with *U*_iso_(H) = 1.2–1.5*U*_eq_(C). The H atoms based on N atoms were located in a difference Fourier map and were refined with a distance restraint of N—H = 0.90Å and with *U*_iso_(H) = 1.2*U*_eq_(N). The H atoms bonded to O atoms were located in a difference Fourier maps and refined with O—H distance restraints of 0.81 (3)Å and with *U*_iso_(H) = 1.3*U*_eq_(O). The H atoms bonded to OW atoms were located in a difference Fourier maps and refined with OW—H = 0.845Å–0.855Å and *U*_iso_(H) = 1–1.2*U*_eq_(OW).

### Crystal data

**Table:**

[Cu(C_14_H_17_N_5_O_3_)_2_(H_2_O)_2_](C_14_H_9_O_5_)_2_	*Z* = 1
*M**_r_* = 1220.66	*F*(000) = 635
Triclinic, *P*1	*D*_x_ = 1.507 Mg m^−^^3^
Hall symbol: -P 1	Mo *K*α radiation, λ = 0.71073 Å
*a* = 8.611 (8) Å	Cell parameters from 11977 reflections
*b* = 12.555 (12) Å	θ = 2.5–27.0°
*c* = 13.436 (12) Å	µ = 0.49 mm^−^^1^
α = 76.222 (10)°	*T* = 295 K
β = 73.299 (10)°	Block, blue
γ = 81.015 (10)°	0.47 × 0.41 × 0.33 mm
*V* = 1345 (2) Å^3^	

### Data collection

**Table:**

Bruker SMART CCD diffractometer	5790 independent reflections
Radiation source: fine-focus sealed tube	4801 reflections with *I* > 2σ(*I*)
graphite	*R*_int_ = 0.086
φ and ω scans	θ_max_ = 27.0°, θ_min_ = 2.5°
Absorption correction: multi-scan (*SADABS*; Sheldrick, 1996)	*h* = −10→10
*T*_min_ = 0.801, *T*_max_ = 0.854	*k* = −15→16
11977 measured reflections	*l* = −17→17

### Refinement

**Table:**

Refinement on *F*^2^	Primary atom site location: structure-invariant direct methods
Least-squares matrix: full	Secondary atom site location: difference Fourier map
*R*[*F*^2^ > 2σ(*F*^2^)] = 0.049	Hydrogen site location: inferred from neighbouring sites
*wR*(*F*^2^) = 0.140	H atoms treated by a mixture of independent and constrained refinement
*S* = 1.04	*w* = 1/[σ^2^(*F*_o_^2^) + (0.080*P*)^2^] where *P* = (*F*_o_^2^ + 2*F*_c_^2^)/3
5790 reflections	(Δ/σ)_max_ < 0.001
397 parameters	Δρ_max_ = 0.56 e Å^−^^3^
3 restraints	Δρ_min_ = −1.01 e Å^−^^3^

### Special details

**Table:**

Geometry. All s.u.'s (except the s.u. in the dihedral angle between two l.s. planes) are estimated using the full covariance matrix. The cell s.u.'s are taken into account individually in the estimation of s.u.'s in distances, angles and torsion angles; correlations between s.u.'s in cell parameters are only used when they are defined by crystal symmetry. An approximate (isotropic) treatment of cell s.u.'s is used for estimating s.u.'s involving l.s. planes.
Refinement. Refinement of *F*^2^ against ALL reflections. The weighted *R*-factor *wR* and goodness of fit *S* are based on *F*^2^, conventional *R*-factors *R* are based on *F*, with *F* set to zero for negative *F*^2^. The threshold expression of *F*^2^ > σ(*F*^2^) is used only for calculating *R*-factors(gt) *etc*. and is not relevant to the choice of reflections for refinement. *R*-factors based on *F*^2^ are statistically about twice as large as those based on *F*, and *R*-factors based on ALL data will be even larger.

### Fractional atomic coordinates and isotropic or equivalent isotropic displacement parameters (Å^2^)

**Table:**

	*x*	*y*	*z*	*U*_iso_*/*U*_eq_	
Cu1	0.0000	0.0000	0.0000	0.03389 (14)	
O1	−0.47101 (18)	0.10445 (10)	0.11352 (13)	0.0351 (3)	
OW1	−0.0750 (3)	−0.05617 (16)	0.20934 (15)	0.0526 (5)	
HW1A	−0.118 (3)	−0.0156 (16)	0.2544 (16)	0.049 (8)*	
HW1B	−0.129 (3)	−0.1115 (15)	0.229 (2)	0.064 (10)*	
O2	−0.21239 (17)	0.08163 (10)	0.02336 (13)	0.0348 (3)	
O3	−0.09237 (18)	−0.13240 (11)	−0.00203 (13)	0.0379 (4)	
O4	0.2629 (2)	−0.06638 (13)	0.62834 (14)	0.0541 (5)	
O5	0.3377 (2)	−0.15031 (13)	0.49121 (16)	0.0545 (5)	
O6	−0.4437 (2)	0.68639 (12)	0.46026 (13)	0.0425 (4)	
H6A	−0.494 (4)	0.745 (2)	0.470 (2)	0.056 (8)*	
O7	−0.3132 (2)	0.78539 (12)	0.30621 (13)	0.0491 (4)	
O8	0.05881 (19)	0.31270 (11)	0.26786 (11)	0.0360 (4)	
N1	−0.3807 (2)	−0.77991 (12)	0.23764 (13)	0.0296 (4)	
H1A	−0.4293	−0.8192	0.2091	0.036*	
H1B	−0.3499	−0.8254	0.2928	0.036*	
N2	−0.4064 (2)	−0.57091 (12)	0.10300 (14)	0.0302 (4)	
N3	−0.2207 (2)	−0.44274 (12)	0.01642 (13)	0.0286 (4)	
N4	−0.48747 (19)	−0.39169 (12)	0.12450 (13)	0.0245 (3)	
N5	−0.56161 (19)	−0.21098 (12)	0.14742 (13)	0.0244 (3)	
C1	−0.3500 (2)	0.04524 (15)	0.07391 (16)	0.0277 (4)	
C2	−0.3694 (2)	−0.07372 (14)	0.08278 (15)	0.0248 (4)	
C3	−0.2405 (2)	−0.15063 (14)	0.04021 (15)	0.0252 (4)	
C4	−0.2879 (2)	−0.25929 (14)	0.05000 (14)	0.0236 (4)	
C5	−0.1829 (2)	−0.34213 (15)	0.00345 (16)	0.0284 (4)	
H5A	−0.0805	−0.3245	−0.0391	0.034*	
C6	−0.3712 (2)	−0.46483 (14)	0.08216 (15)	0.0240 (4)	
C7	−0.4444 (2)	−0.28933 (14)	0.10703 (14)	0.0224 (4)	
C8	−0.5211 (2)	−0.10795 (14)	0.13354 (15)	0.0255 (4)	
H8A	−0.6022	−0.0562	0.1604	0.031*	
C9	−0.7306 (2)	−0.23875 (16)	0.20318 (16)	0.0302 (4)	
H9A	−0.7622	−0.2865	0.1668	0.036*	
H9B	−0.8038	−0.1717	0.1998	0.036*	
C10	−0.7491 (3)	−0.2952 (2)	0.31814 (18)	0.0485 (6)	
H10A	−0.8601	−0.3112	0.3504	0.073*	
H10B	−0.7202	−0.2477	0.3550	0.073*	
H10C	−0.6789	−0.3626	0.3220	0.073*	
C11	−0.2860 (3)	−0.65883 (14)	0.06486 (16)	0.0295 (4)	
H11A	−0.1919	−0.6267	0.0138	0.035*	
H11B	−0.3332	−0.6995	0.0296	0.035*	
C12	−0.2338 (2)	−0.73607 (15)	0.15641 (16)	0.0301 (4)	
H12A	−0.1598	−0.7964	0.1314	0.036*	
H12B	−0.1773	−0.6972	0.1877	0.036*	
C13	−0.4992 (3)	−0.68931 (16)	0.27633 (16)	0.0324 (4)	
H13A	−0.4496	−0.6494	0.3109	0.039*	
H13B	−0.5941	−0.7199	0.3278	0.039*	
C14	−0.5504 (2)	−0.61120 (15)	0.18377 (18)	0.0332 (5)	
H14A	−0.6098	−0.6492	0.1536	0.040*	
H14B	−0.6218	−0.5496	0.2083	0.040*	
C15	0.1449 (3)	0.12351 (18)	0.30272 (19)	0.0443 (6)	
H15A	0.1362	0.1210	0.2359	0.053*	
C16	0.2015 (3)	0.03110 (18)	0.36527 (19)	0.0476 (6)	
H16A	0.2319	−0.0340	0.3400	0.057*	
C17	0.2144 (3)	0.03297 (15)	0.46562 (16)	0.0312 (4)	
C18	0.1694 (3)	0.13108 (16)	0.50118 (17)	0.0350 (5)	
H18A	0.1772	0.1336	0.5682	0.042*	
C19	0.1130 (3)	0.22545 (16)	0.43918 (16)	0.0328 (5)	
H19A	0.0838	0.2909	0.4638	0.039*	
C20	0.1008 (2)	0.22064 (15)	0.34007 (16)	0.0283 (4)	
C21	−0.0382 (2)	0.40359 (15)	0.30085 (16)	0.0284 (4)	
C22	−0.0043 (3)	0.50373 (17)	0.23318 (17)	0.0351 (5)	
H22A	0.0823	0.5074	0.1726	0.042*	
C23	−0.1005 (3)	0.59877 (16)	0.25639 (17)	0.0333 (5)	
H23A	−0.0785	0.6662	0.2107	0.040*	
C24	−0.2295 (2)	0.59424 (15)	0.34728 (16)	0.0288 (4)	
C25	−0.2633 (3)	0.49235 (17)	0.41362 (17)	0.0365 (5)	
H25A	−0.3501	0.4883	0.4741	0.044*	
C26	−0.1687 (3)	0.39667 (16)	0.39035 (18)	0.0354 (5)	
H26A	−0.1928	0.3287	0.4344	0.043*	
C27	0.2781 (3)	−0.06920 (16)	0.53302 (19)	0.0375 (5)	
C28	−0.3324 (3)	0.69794 (16)	0.36871 (17)	0.0318 (4)	

### Atomic displacement parameters (Å^2^)

**Table:**

	*U*^11^	*U*^22^	*U*^33^	*U*^12^	*U*^13^	*U*^23^
Cu1	0.0228 (2)	0.02319 (19)	0.0572 (3)	−0.00498 (13)	−0.00532 (17)	−0.01570 (16)
O1	0.0281 (8)	0.0259 (7)	0.0542 (10)	0.0003 (5)	−0.0070 (7)	−0.0204 (6)
OW1	0.0558 (12)	0.0515 (11)	0.0439 (10)	−0.0037 (9)	−0.0031 (9)	−0.0101 (8)
O2	0.0277 (8)	0.0232 (7)	0.0524 (9)	−0.0036 (5)	−0.0055 (7)	−0.0109 (6)
O3	0.0254 (8)	0.0276 (7)	0.0602 (10)	−0.0082 (6)	0.0024 (7)	−0.0212 (6)
O4	0.0749 (14)	0.0403 (9)	0.0428 (10)	0.0150 (8)	−0.0237 (9)	−0.0033 (7)
O5	0.0603 (12)	0.0321 (8)	0.0657 (12)	0.0172 (8)	−0.0160 (9)	−0.0140 (8)
O6	0.0433 (10)	0.0307 (8)	0.0410 (9)	0.0085 (7)	0.0017 (7)	−0.0068 (6)
O7	0.0612 (12)	0.0294 (8)	0.0451 (10)	0.0032 (7)	−0.0046 (8)	−0.0017 (7)
O8	0.0431 (9)	0.0331 (7)	0.0270 (7)	0.0107 (6)	−0.0080 (6)	−0.0073 (6)
N1	0.0354 (10)	0.0264 (8)	0.0285 (9)	−0.0012 (7)	−0.0120 (7)	−0.0049 (6)
N2	0.0260 (9)	0.0195 (7)	0.0405 (10)	−0.0045 (6)	−0.0013 (7)	−0.0052 (7)
N3	0.0262 (9)	0.0249 (8)	0.0325 (9)	−0.0048 (6)	0.0014 (7)	−0.0111 (6)
N4	0.0223 (8)	0.0214 (7)	0.0305 (8)	−0.0047 (6)	−0.0057 (7)	−0.0064 (6)
N5	0.0206 (8)	0.0229 (7)	0.0284 (8)	−0.0028 (6)	−0.0022 (6)	−0.0075 (6)
C1	0.0299 (11)	0.0244 (9)	0.0333 (10)	−0.0025 (7)	−0.0125 (8)	−0.0094 (7)
C2	0.0261 (10)	0.0206 (8)	0.0286 (10)	−0.0028 (7)	−0.0065 (8)	−0.0073 (7)
C3	0.0222 (10)	0.0242 (9)	0.0305 (10)	−0.0056 (7)	−0.0038 (8)	−0.0095 (7)
C4	0.0217 (9)	0.0233 (8)	0.0268 (10)	−0.0038 (7)	−0.0046 (7)	−0.0081 (7)
C5	0.0231 (10)	0.0275 (9)	0.0336 (10)	−0.0059 (7)	−0.0013 (8)	−0.0099 (8)
C6	0.0258 (10)	0.0223 (8)	0.0254 (9)	−0.0024 (7)	−0.0084 (8)	−0.0054 (7)
C7	0.0226 (10)	0.0224 (8)	0.0236 (9)	−0.0028 (7)	−0.0071 (7)	−0.0057 (7)
C8	0.0246 (10)	0.0232 (8)	0.0288 (10)	−0.0004 (7)	−0.0043 (8)	−0.0103 (7)
C9	0.0194 (10)	0.0322 (10)	0.0376 (11)	−0.0053 (7)	0.0010 (8)	−0.0128 (8)
C10	0.0492 (15)	0.0577 (14)	0.0351 (13)	−0.0167 (11)	0.0006 (11)	−0.0099 (11)
C11	0.0342 (11)	0.0226 (9)	0.0317 (10)	−0.0034 (8)	−0.0045 (8)	−0.0100 (7)
C12	0.0269 (11)	0.0298 (9)	0.0366 (11)	0.0013 (7)	−0.0079 (9)	−0.0156 (8)
C13	0.0292 (11)	0.0329 (10)	0.0332 (11)	−0.0052 (8)	−0.0018 (9)	−0.0099 (8)
C14	0.0221 (10)	0.0242 (9)	0.0490 (13)	−0.0046 (7)	−0.0026 (9)	−0.0060 (8)
C15	0.0632 (17)	0.0390 (12)	0.0325 (12)	0.0083 (11)	−0.0155 (11)	−0.0156 (9)
C16	0.0695 (18)	0.0336 (11)	0.0399 (13)	0.0162 (11)	−0.0149 (12)	−0.0205 (10)
C17	0.0306 (11)	0.0259 (9)	0.0324 (11)	0.0042 (8)	−0.0044 (8)	−0.0062 (8)
C18	0.0450 (13)	0.0317 (10)	0.0303 (11)	0.0020 (9)	−0.0133 (9)	−0.0093 (8)
C19	0.0404 (12)	0.0262 (9)	0.0315 (11)	0.0047 (8)	−0.0084 (9)	−0.0116 (8)
C20	0.0242 (10)	0.0294 (9)	0.0289 (10)	0.0016 (7)	−0.0052 (8)	−0.0061 (7)
C21	0.0275 (11)	0.0298 (9)	0.0292 (10)	0.0026 (8)	−0.0108 (8)	−0.0077 (8)
C22	0.0331 (12)	0.0378 (11)	0.0262 (10)	0.0014 (9)	−0.0009 (9)	−0.0023 (8)
C23	0.0351 (12)	0.0288 (10)	0.0304 (11)	−0.0028 (8)	−0.0052 (9)	0.0004 (8)
C24	0.0291 (11)	0.0296 (9)	0.0286 (10)	−0.0003 (8)	−0.0092 (8)	−0.0072 (8)
C25	0.0306 (12)	0.0339 (10)	0.0348 (12)	0.0008 (8)	0.0018 (9)	−0.0027 (9)
C26	0.0335 (12)	0.0270 (10)	0.0370 (12)	−0.0007 (8)	−0.0024 (9)	0.0004 (8)
C27	0.0362 (12)	0.0276 (10)	0.0445 (13)	0.0034 (8)	−0.0104 (10)	−0.0036 (9)
C28	0.0333 (12)	0.0303 (10)	0.0327 (11)	0.0004 (8)	−0.0106 (9)	−0.0081 (8)

### Geometric parameters (Å, °)

**Table:**

Cu1—O2	1.932 (2)	C9—C10	1.512 (3)
Cu1—O2^i^	1.932 (2)	C9—H9A	0.9700
Cu1—O3^i^	1.9605 (19)	C9—H9B	0.9700
Cu1—O3	1.9605 (19)	C10—H10A	0.9600
O1—C1	1.245 (3)	C10—H10B	0.9600
OW1—HW1A	0.850 (10)	C10—H10C	0.9600
OW1—HW1B	0.845 (10)	C11—C12	1.506 (3)
O2—C1	1.278 (3)	C11—H11A	0.9700
O3—C3	1.271 (2)	C11—H11B	0.9700
O4—C27	1.258 (3)	C12—H12A	0.9700
O5—C27	1.249 (3)	C12—H12B	0.9700
O6—C28	1.317 (3)	C13—C14	1.517 (3)
O6—H6A	0.81 (3)	C13—H13A	0.9700
O7—C28	1.215 (3)	C13—H13B	0.9700
O8—C21	1.388 (2)	C14—H14A	0.9700
O8—C20	1.395 (2)	C14—H14B	0.9700
N1—C13	1.490 (3)	C15—C16	1.373 (3)
N1—C12	1.494 (3)	C15—C20	1.386 (3)
N1—H1A	0.9000	C15—H15A	0.9300
N1—H1B	0.9000	C16—C17	1.390 (3)
N2—C6	1.358 (3)	C16—H16A	0.9300
N2—C14	1.461 (3)	C17—C18	1.386 (3)
N2—C11	1.470 (3)	C17—C27	1.510 (3)
N3—C5	1.312 (3)	C18—C19	1.384 (3)
N3—C6	1.370 (3)	C18—H18A	0.9300
N4—C7	1.341 (3)	C19—C20	1.381 (3)
N4—C6	1.343 (2)	C19—H19A	0.9300
N5—C8	1.348 (3)	C21—C22	1.382 (3)
N5—C7	1.382 (2)	C21—C26	1.386 (3)
N5—C9	1.486 (3)	C22—C23	1.388 (3)
C1—C2	1.501 (3)	C22—H22A	0.9300
C2—C8	1.371 (3)	C23—C24	1.391 (3)
C2—C3	1.434 (3)	C23—H23A	0.9300
C3—C4	1.450 (3)	C24—C25	1.393 (3)
C4—C7	1.405 (3)	C24—C28	1.495 (3)
C4—C5	1.408 (3)	C25—C26	1.390 (3)
C5—H5A	0.9300	C25—H25A	0.9300
C8—H8A	0.9300	C26—H26A	0.9300
			
O2—Cu1—O2^i^	180.00 (9)	N2—C11—H11B	109.6
O2—Cu1—O3^i^	88.51 (9)	C12—C11—H11B	109.6
O2^i^—Cu1—O3^i^	91.49 (9)	H11A—C11—H11B	108.1
O2—Cu1—O3	91.49 (9)	N1—C12—C11	109.21 (17)
O2^i^—Cu1—O3	88.51 (9)	N1—C12—H12A	109.8
O3^i^—Cu1—O3	180.00 (9)	C11—C12—H12A	109.8
HW1A—OW1—HW1B	106.0 (15)	N1—C12—H12B	109.8
C1—O2—Cu1	128.03 (13)	C11—C12—H12B	109.8
C3—O3—Cu1	124.09 (12)	H12A—C12—H12B	108.3
C28—O6—H6A	111 (2)	N1—C13—C14	109.85 (18)
C21—O8—C20	121.82 (16)	N1—C13—H13A	109.7
C13—N1—C12	111.43 (15)	C14—C13—H13A	109.7
C13—N1—H1A	109.3	N1—C13—H13B	109.7
C12—N1—H1A	109.3	C14—C13—H13B	109.7
C13—N1—H1B	109.3	H13A—C13—H13B	108.2
C12—N1—H1B	109.3	N2—C14—C13	109.62 (17)
H1A—N1—H1B	108.0	N2—C14—H14A	109.7
C6—N2—C14	122.40 (16)	C13—C14—H14A	109.7
C6—N2—C11	122.31 (17)	N2—C14—H14B	109.7
C14—N2—C11	113.87 (15)	C13—C14—H14B	109.7
C5—N3—C6	115.40 (16)	H14A—C14—H14B	108.2
C7—N4—C6	115.62 (17)	C16—C15—C20	119.3 (2)
C8—N5—C7	118.91 (17)	C16—C15—H15A	120.3
C8—N5—C9	120.33 (15)	C20—C15—H15A	120.3
C7—N5—C9	120.74 (15)	C15—C16—C17	121.2 (2)
O1—C1—O2	123.21 (17)	C15—C16—H16A	119.4
O1—C1—C2	118.03 (19)	C17—C16—H16A	119.4
O2—C1—C2	118.72 (17)	C18—C17—C16	118.30 (18)
C8—C2—C3	119.53 (17)	C18—C17—C27	121.2 (2)
C8—C2—C1	116.70 (16)	C16—C17—C27	120.53 (19)
C3—C2—C1	123.76 (18)	C19—C18—C17	121.5 (2)
O3—C3—C2	126.19 (17)	C19—C18—H18A	119.3
O3—C3—C4	118.81 (16)	C17—C18—H18A	119.3
C2—C3—C4	114.99 (17)	C20—C19—C18	118.79 (18)
C7—C4—C5	114.89 (17)	C20—C19—H19A	120.6
C7—C4—C3	121.86 (16)	C18—C19—H19A	120.6
C5—C4—C3	123.25 (18)	C19—C20—C15	120.88 (18)
N3—C5—C4	124.03 (19)	C19—C20—O8	123.37 (18)
N3—C5—H5A	118.0	C15—C20—O8	115.51 (19)
C4—C5—H5A	118.0	C22—C21—C26	120.83 (18)
N4—C6—N2	117.63 (18)	C22—C21—O8	115.21 (19)
N4—C6—N3	126.45 (16)	C26—C21—O8	123.78 (18)
N2—C6—N3	115.89 (16)	C21—C22—C23	119.5 (2)
N4—C7—N5	117.44 (18)	C21—C22—H22A	120.3
N4—C7—C4	123.08 (16)	C23—C22—H22A	120.3
N5—C7—C4	119.48 (16)	C22—C23—C24	120.70 (19)
N5—C8—C2	124.93 (16)	C22—C23—H23A	119.6
N5—C8—H8A	117.5	C24—C23—H23A	119.6
C2—C8—H8A	117.5	C25—C24—C23	118.96 (18)
N5—C9—C10	112.82 (17)	C25—C24—C28	121.8 (2)
N5—C9—H9A	109.0	C23—C24—C28	119.23 (18)
C10—C9—H9A	109.0	C26—C25—C24	120.7 (2)
N5—C9—H9B	109.0	C26—C25—H25A	119.7
C10—C9—H9B	109.0	C24—C25—H25A	119.7
H9A—C9—H9B	107.8	C21—C26—C25	119.30 (19)
C9—C10—H10A	109.5	C21—C26—H26A	120.4
C9—C10—H10B	109.5	C25—C26—H26A	120.4
H10A—C10—H10B	109.5	O5—C27—O4	125.2 (2)
C9—C10—H10C	109.5	O5—C27—C17	118.0 (2)
H10A—C10—H10C	109.5	O4—C27—C17	116.79 (19)
H10B—C10—H10C	109.5	O7—C28—O6	123.32 (19)
N2—C11—C12	110.25 (17)	O7—C28—C24	122.1 (2)
N2—C11—H11A	109.6	O6—C28—C24	114.58 (17)
C12—C11—H11A	109.6		
			
O3^i^—Cu1—O2—C1	−148.18 (18)	C1—C2—C8—N5	178.93 (17)
O3—Cu1—O2—C1	31.82 (18)	C8—N5—C9—C10	−99.3 (2)
O2—Cu1—O3—C3	−22.22 (17)	C7—N5—C9—C10	82.1 (2)
O2^i^—Cu1—O3—C3	157.78 (17)	C6—N2—C11—C12	109.8 (2)
Cu1—O2—C1—O1	156.75 (15)	C14—N2—C11—C12	−56.9 (2)
Cu1—O2—C1—C2	−25.6 (3)	C13—N1—C12—C11	−58.2 (2)
O1—C1—C2—C8	0.6 (3)	N2—C11—C12—N1	55.9 (2)
O2—C1—C2—C8	−177.16 (17)	C12—N1—C13—C14	58.3 (2)
O1—C1—C2—C3	179.61 (18)	C6—N2—C14—C13	−110.5 (2)
O2—C1—C2—C3	1.9 (3)	C11—N2—C14—C13	56.2 (2)
Cu1—O3—C3—C2	9.3 (3)	N1—C13—C14—N2	−55.5 (2)
Cu1—O3—C3—C4	−169.20 (13)	C20—C15—C16—C17	0.5 (4)
C8—C2—C3—O3	−174.87 (19)	C15—C16—C17—C18	−0.4 (4)
C1—C2—C3—O3	6.1 (3)	C15—C16—C17—C27	−179.5 (2)
C8—C2—C3—C4	3.7 (3)	C16—C17—C18—C19	0.0 (4)
C1—C2—C3—C4	−175.33 (17)	C27—C17—C18—C19	179.1 (2)
O3—C3—C4—C7	172.18 (17)	C17—C18—C19—C20	0.4 (3)
C2—C3—C4—C7	−6.5 (3)	C18—C19—C20—C15	−0.3 (3)
O3—C3—C4—C5	−8.1 (3)	C18—C19—C20—O8	−174.38 (19)
C2—C3—C4—C5	173.23 (18)	C16—C15—C20—C19	−0.1 (4)
C6—N3—C5—C4	−1.7 (3)	C16—C15—C20—O8	174.4 (2)
C7—C4—C5—N3	−4.3 (3)	C21—O8—C20—C19	−28.0 (3)
C3—C4—C5—N3	176.01 (19)	C21—O8—C20—C15	157.6 (2)
C7—N4—C6—N2	176.26 (17)	C20—O8—C21—C22	147.58 (19)
C7—N4—C6—N3	−6.0 (3)	C20—O8—C21—C26	−37.3 (3)
C14—N2—C6—N4	−11.6 (3)	C26—C21—C22—C23	1.3 (3)
C11—N2—C6—N4	−177.18 (17)	O8—C21—C22—C23	176.50 (19)
C14—N2—C6—N3	170.41 (17)	C21—C22—C23—C24	0.5 (3)
C11—N2—C6—N3	4.9 (3)	C22—C23—C24—C25	−1.5 (3)
C5—N3—C6—N4	7.4 (3)	C22—C23—C24—C28	−178.9 (2)
C5—N3—C6—N2	−174.85 (18)	C23—C24—C25—C26	0.7 (3)
C6—N4—C7—N5	178.69 (15)	C28—C24—C25—C26	178.1 (2)
C6—N4—C7—C4	−1.1 (3)	C22—C21—C26—C25	−2.0 (3)
C8—N5—C7—N4	178.36 (17)	O8—C21—C26—C25	−176.78 (19)
C9—N5—C7—N4	−3.0 (2)	C24—C25—C26—C21	0.9 (3)
C8—N5—C7—C4	−1.9 (3)	C18—C17—C27—O5	−170.2 (2)
C9—N5—C7—C4	176.77 (16)	C16—C17—C27—O5	8.9 (3)
C5—C4—C7—N4	5.8 (3)	C18—C17—C27—O4	11.6 (3)
C3—C4—C7—N4	−174.52 (17)	C16—C17—C27—O4	−169.3 (2)
C5—C4—C7—N5	−173.98 (16)	C25—C24—C28—O7	−173.0 (2)
C3—C4—C7—N5	5.7 (3)	C23—C24—C28—O7	4.4 (3)
C7—N5—C8—C2	−0.9 (3)	C25—C24—C28—O6	7.3 (3)
C9—N5—C8—C2	−179.57 (18)	C23—C24—C28—O6	−175.3 (2)
C3—C2—C8—N5	−0.1 (3)		

Symmetry codes: (i) −*x*, −*y*, −*z*.

### Hydrogen-bond geometry (Å, °)

**Table:**

*D*—H···*A*	*D*—H	H···*A*	*D*···*A*	*D*—H···*A*
OW1—HW1A···O4^ii^	0.85 (1)	2.08 (1)	2.921 (3)	169 (2)
OW1—HW1B···O7^iii^	0.85 (1)	2.08 (3)	2.892 (3)	161 (2)
O6—H6A···O5^iv^	0.81 (3)	1.81 (3)	2.582 (3)	160 (3)
N1—H1A···O1^iii^	0.90	1.91	2.783 (3)	162
N1—H1B···O4^v^	0.90	1.73	2.604 (3)	164

Symmetry codes: (ii) −*x*, −*y*, −*z*+1; (iii) *x*, *y*−1, *z*; (iv) *x*−1, *y*+1, *z*; (v) −*x*, −*y*−1, −*z*+1.

## References

[bb1] Bruker (2001). *SMART* and *SAINT* Bruker AXS Inc., Madison Wisconsion, USA.

[bb2] Mizuki, Y., Fujiwara, I. & Yamaguchi, T. (1996). *J. Antimicrob. Chemother. Suppl. A*, **37**, 41–45. 10.1093/jac/37.suppl_a.41 8737124

[bb3] Sheldrick, G. M. (1996). *SADABS* University of Göttingen, Germany.

[bb4] Sheldrick, G. M. (2008). *Acta Cryst.* A**64**, 112–122. 10.1107/S0108767307043930 18156677

